# Analgesic Effects of Ultrasound‐Guided Iliohypogastric/Ilioinguinal Nerve Block Combined with Lateral Femoral Cutaneous Nerve Block in Total Hip Arthroplasty *via* Direct Anterior Approach: A Retrospective Cohort Study

**DOI:** 10.1111/os.12795

**Published:** 2021-03-31

**Authors:** Qiuru Wang, Yong Yang, Zhouyuan Yang, Yunlian Hu, Xin Zhao, Changjun Chen, Pengde Kang

**Affiliations:** ^1^ Department of Orthopaedic Surgery, West China Hospital Sichuan University Chengdu China; ^2^ Department of Orthopaedic Surgery Karamay Municipal People's Hospital Karamay China; ^3^ Department of General Surgery Yongchuan Hospital of Traditional Chinese Medicine Yongchuan China

**Keywords:** Direct anterior approach, Enhanced recovery, Pain, Peripheral nerve blocks, Total hip arthroplasty

## Abstract

**Objective:**

This study aimed to explore the efficacy and safety of the combination of lateral femoral cutaneous nerve blocks (LFCNB) and iliohypogastric/ilioinguinal nerve blocks (IHINB) on postoperative pain and functional outcomes after total hip arthroplasty (THA) *via* the direct anterior approach (DAA).

**Methods:**

In this retrospective cohort study, patients undergoing THA *via* the DAA between January 2019 and November 2019 were stratified into two groups based on their date of admission. Sixty‐seven patients received LFCNB and IHINB along with periarticular infiltration analgesia (PIA) (nerve block group), and 75 patients received PIA alone (control group). The outcomes included postoperative morphine consumption, postoperative pain assessed using the visual analogue scale (VAS), the QoR‐15 score, and functional recovery measured as quadriceps strength, time to first straight leg rise, daily ambulation distance, and duration of hospitalization. The Oxford hip score and the UCLA activity level rating were assessed at 1 and 3 months after surgery. In addition, postoperative complications were recorded. Patients were also compared based on the type of incision used during surgery (traditional longitudinal or “bikini” incision).

**Results:**

Patients in the nerve block group showed significantly lower postoperative morphine consumption, lower resting VAS scores within 12 h postoperatively, lower VAS scores during motion within 24 h postoperatively, and better QoR‐15 scores on postoperative day 1. These patients also showed significantly better functional recovery during hospitalization. At 1‐month and 3‐month outpatient follow up, the two groups showed no significant differences in Oxford hip score or UCLA activity level rating. There were no significant differences in the incidence of postoperative complications. Similar results were observed when patients were stratified by type of incision, except that the duration of hospitalization was similar.

**Conclusion:**

Compared to PIA alone, a combination of LFCNB and IHINB along with PIA can improve early pain relief, reduce morphine consumption, and accelerate functional recovery, without increasing complications after THA *via* the DAA.

## Graphical abstract

The location of the iliohypogastric nerve (IHN), the ilioinguinal nerve (IIN), and the lateral femoral cutaneous nerve (LFCN) (A). Schematic diagram of the location of the nerve block (B). The upper block is the iliohypogastric/ilioinguinal nerve block (IHINB) and the lower block is the lateral femoral cutaneous nerve block (LFCNB). The blue box shows the position of the ultrasonic transducer and the symbol x shows the needle insertion point. ASIS, anterior superior iliac spine. Schematic diagram of the IHINB (A). The high‐frequency linear‐array ultrasonic transducer was placed perpendicular to the inguinal ligament, with the lower end of the transducer at the ASIS and the upper end facing the umbilicus. The needle was inserted under the transducer from lateral to medial side in‐plane. Schematic diagram of the LFCNB (B). The transducer was placed on the inguinal ligament, with the upper end above the ASIS and the lower end pointing to the pubic symphysis. Then the transducer was moved along the inguinal ligament slowly inward and downward until the LFCN was detected. The needle was inserted from lateral to medial side in‐plane.

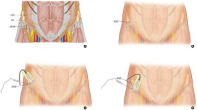

## Introduction

Arthroplasty is one of the most painful orthopaedic surgical procedures^1^. More than half of total hip arthroplasty (THA) patients suffer moderate to severe pain after surgery^2^. Ineffective pain management often delays recovery in THA patients, and the lack of exercise due to postoperative pain can lead to immobility‐related complications, such as venous thromboembolism (VTE) and arthrofibrosis^3^. To enhance recovery after THA, pain is managed using numerous multimodal analgesia methods, such as oral analgesics, peripheral nerve blocks, and periarticular infiltration analgesia (PIA)^4^, ^5^, ^6^, ^7^.

Currently, procedures involving peripheral nerve blocks are critical for perioperative multimodal analgesia in THA. Blocks of peripheral nerves, including femoral, fascia iliaca, and lumbar plexus nerves, can improve analgesia but are also associated with motor blockade and falls^5^, ^8^. Purely sensory blocking is the priority of early rehabilitation exercises, so it is essential to identify nerve blocks that can aid in the functional recovery of THA patients.

The lateral femoral cutaneous nerve is a sensory branch from the lumbar plexus and supplies parts of the lateral and anterior upper thigh^9^, ^10^, ^11^, ^12^. Lateral femoral cutaneous nerve block (LFCNB) has been used to reduce pain after posterior approach THA, but its analgesic effect remains controversial^13^, ^14^. LFCNB was reported to reduce movement‐related pain in patients with moderate to severe pain after THA, although combining LFCNB with paracetamol and ibuprofen after THA *via* the posterior approach did not increase the analgesic effect^13^, ^14^. The iliohypogastric and the ilioinguinal nerves also extend from the lumbar plexus and supply the lateral buttock, the inguinal area, and the medial upper thigh^15^. Currently, iliohypogastric/ilioinguinal nerve block (IHINB) is used mainly to achieve postoperative analgesia for surgeries performed in the inguinal or perineal region, without reducing the muscle strength of lower limbs^15^, ^16^, ^17^, ^18^.

**Fig. 1:**
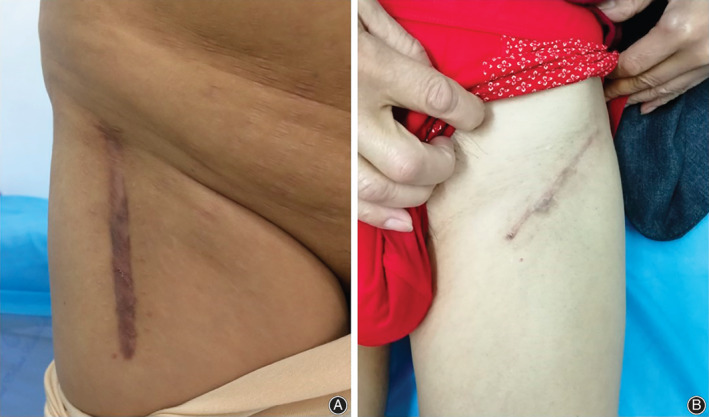
The location of traditional longitudinal incision (A) and “bikini” incision (B).

The direct anterior approach (DAA) is a minimally invasive surgical approach of THA. There are two commonly used surgical incisions in THA *via* the DAA. The traditional longitudinal incision (Fig. 1A) is 8–10 cm long, and is made laterally and distally to the anterosuperior iliac spine and directed towards the fibular head, while the oblique “bikini” incision (Fig. 1B) is 8–10 cm long and centered in the inguinal crease^19^, ^20^, ^21^. Wound pain can contribute to postoperative pain^22^, ^23^. The surgical sites of both incisions are located in the area supplied by the iliohypogastric/ilioinguinal nerve and the lateral femoral cutaneous nerve. Therefore, we hypothesize that, compared to the analgesic effects of PIA alone, a combination of LFCNB and IHINB along with PIA may improve pain relief and functional recovery in patients undergoing THA *via* the DAA. Using a retrospective approach, the purpose of this study was to evaluate the efficacy and safety of these two peripheral nerve blocks for pain management after THA *via* the DAA. At the same time, we aimed to compare patients who underwent the traditional longitudinal or “bikini” incision separately during analysis.

## Methods and Materials

This study was approved by the Clinical Trials and Biomedical Ethics Committee of Sichuan University West China Hospital. Before surgery, written informed consent was obtained from all participants to allow their anonymized clinical data to be analyzed and published for research purposes.

### Patients

Inclusion criteria of patients included: (i) patients undergoing primary, unilateral THA *via* the DAA using longitudinal or “bikini” incisions between January 2019 and November 2019; (ii) patients that received only PIA or received LFCNB + IHINB combined with PIA; and (iii) patients that had an American Society of Anesthesiologists (ASA) functional status of I–III.

Exclusion criteria included: (i) body mass index (BMI) >30 kg/m^2^; (ii) previous hip open surgery; (iii) intolerance of general anesthesia; (iv) known allergies to the drugs used in this study; (v) recognized neuromuscular disorders; or (vi) patient unable to communicate verbally.

A total of 179 patients were screened for eligibility. Patients were stratified into two groups (control or nerve block) based on their date of admission. Patients admitted before 1 July 2019 received only PIA (control group), while patients admitted after 1 July 2019 received LFCNB + IHINB combined with PIA (nerve block group). Thus, patients were not assigned to these groups based on clinicodemographic characteristics.

### Perioperative Analgesia and Management

On the day before the surgical procedure, celecoxib (200 mg) was administered twice to all patients as a preemptive analgesic. Nerve blocks, in the form of a local anesthetic consisting of 0.33% ropivacaine and 2.0 μg/mL of epinephrine, were administered to patients in the supine position 30 min before general anesthesia.

**Fig. 2:**
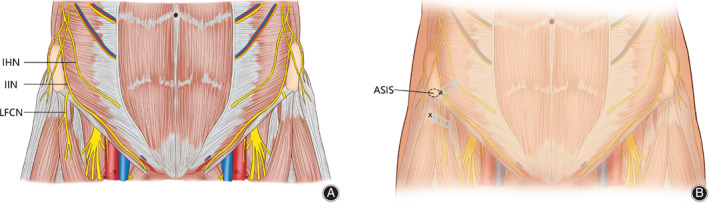
The location of the iliohypogastric nerve (IHN), the ilioinguinal nerve (IIN), and the lateral femoral cutaneous nerve (LFCN) (A). Schematic diagram of the location of nerve block (B). The upper block is the iliohypogastric/ilioinguinal nerve block (IHINB) and the lower block is the lateral femoral cutaneous nerve block (LFCNB). The blue box is the position of the ultrasonic transducer and the symbol x is the needle insertion point. ASIS, anterior superior iliac spine.

**Fig. 3:**
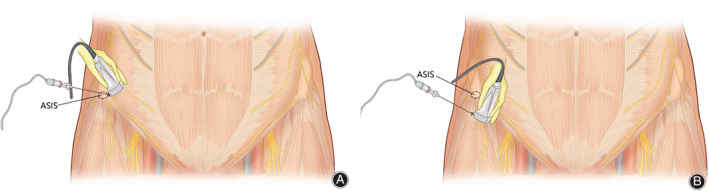
Schematic diagram of the iliohypogastric/ilioinguinal nerve block (IHINB) (A). The high‐frequency linear‐array ultrasonic transducer was placed perpendicular to the inguinal ligament, with the lower end of the transducer at the ASIS and the upper end facing the umbilicus. The needle was inserted under the transducer from lateral to medial side in‐plane. Schematic diagram of the lateral femoral cutaneous nerve block (LFCNB) (B). The transducer was placed on the inguinal ligament, with the upper end above the ASIS and the lower end pointing to the pubic symphysis. Then the transducer was moved along the inguinal ligament slowly inward and downward until the lateral femoral cutaneous nerve (LFCN) was detected. The needle was inserted from lateral to medial side in‐plane.

**Fig. 4:**
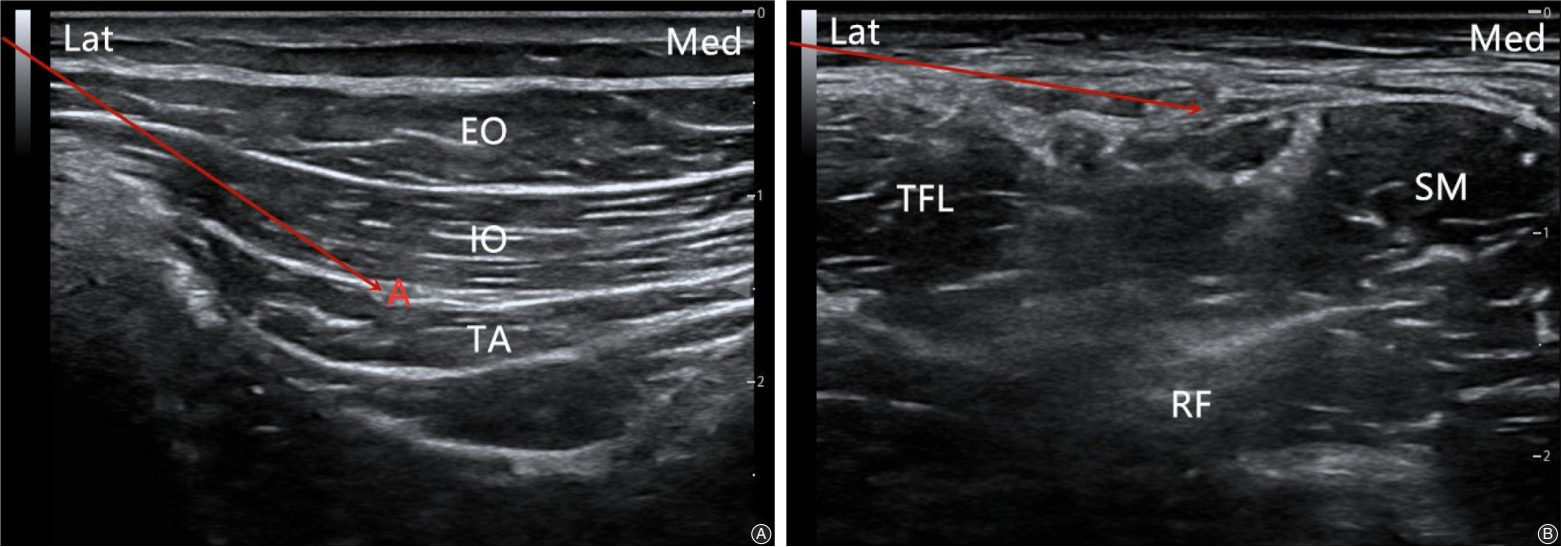
The ultrasound images of IHINB (A) and the lateral femoral cutaneous nerve block (LFCNB) (B). The line shows the needle insertion point. EO, external oblique; IO, internal oblique; RF, rectus femoris; SM, sartorius muscle; TA, transverse abdominis; TFL, tensor fasciae latae.

The IHINB was performed under local anesthesia (5 mL) using a high‐frequency linear‐array ultrasonic transducer (Anesus ME7, Mindray, Shenzhen, China). The area between the umbilicus and the anterior superior iliac spine was swept, and a local anesthetic was injected after visualization of the iliohypogastric and ilioinguinal nerves. All injections were administered in‐plane with a 21‐gauge, 100‐mm needle (Pajunk, Geisingen, Germany). In the case of LFCNB, the abovementioned ultrasonic transducer technique was used to identify the lateral femoral cutaneous nerve between the origin of the sartorius and the tensor fasciae latae muscle. A local anesthetic (5 mL) was injected to cover the lateral cutaneous aspect of the hip. The key procedures for these two techniques are illustrated in Figs 2, 3, 4.

All surgeries were conducted under general anesthesia. Patients were given anesthetics intravenously (midazolam 2 mg, propofol 2 mg/kg, sufentanil 0.3 μg/kg, cisatracurium 0.2 mg/kg) after pure oxygen inhalation. Then, patients were intubated and given inhaled anesthetics (sevoflurane, 1–1.5 MAC). All surgical procedures on patients in this study were performed by the same surgeon from our institution, whose learning curve met the required skill level as defined by de Steiger *et al*.^24^. Corail or TRI‐LOCK stems and PINNACLE Cups (DePuy Synthes, New Brunswick, NJ, USA) were used in all patients. Before the wound was sutured, PIA (20 mL of 0.33% ropivacaine and 2.0 μg/mL of epinephrine) was administered *via* multiple doses injected into tissues around the hip and the incision site. All incisions were closed using the same technique by another surgeon (first assistant).

After awakening from general anesthesia, patients were sent to a ward and an ice compress was applied around the incision. Celecoxib (200 mg) was administered twice daily to control postoperative pain. If the patient was unable to tolerate the pain, morphine hydrochloride (10 mg) was injected subcutaneously as rescue analgesia. Enoxaparin (0.2 mL) was administered 12 h after surgery, followed by additional doses (0.4 mL) every 24 h until discharge to prevent VTE. Rivaroxaban (10 mg) was also administered once a day for 2 weeks after discharge to continue to prevent VTE. During postoperative hospitalization, all patients had to walk with a walking aid. After discharge, patients were required to return to the outpatient department of our institution for follow up at 1 and 3 months after surgery.

### Outcome Measures

#### Patient Characteristics

The following patient characteristics were recorded at admission: age, gender, BMI, preoperative pain scores during motion, quadriceps strength, Oxford hip score, UCLA activity level rating, and ASA functional status.

### Postoperative Outcomes

#### Postoperative Morphine Consumption

The total consumption of supplementary morphine hydrochloride was recorded every day after surgery, and the total morphine consumption during hospitalization was also recorded. The decisions regarding use of morphine hydrochloride were made by a pharmacist, who was part of the pain management team. The pharmacist was typically unaware of the treatment group.

#### Postoperative Pain

Postoperative pain at rest and during motion (hip flexion of 45°) was measured using a visual analogue scale (VAS) score^25^. The scale ranged from 0 to 10, where 0 indicates no pain and 10 indicates extreme pain. A pain score of 1–3 was considered mild pain. We measured pain at rest at 2, 6, 12, 24, and 36 h after surgery and at discharge, as well as pain during motion at 6, 12, 24, and 36 h after surgery and at discharge. VAS pain scores were also assessed at 3‐month outpatient follow‐up.

#### Postoperative Recovery Quality

Postoperative recovery quality of patients was measured using the QoR‐15 quality of recovery score^26^ on postoperative days 1 and 2. The QoR‐15 score ranges from 0 to 150, with higher scores suggesting better quality of postoperative recovery.

#### Postoperative Functional Recovery

Postoperative functional recovery of patients was measured by quadriceps strength, time to first straight leg rise, daily ambulation distance during hospitalization, and the duration of hospitalization. In addition, the Oxford hip score^27^ and the UCLA activity level rating^28^ were assessed at 1 and 3 months after surgery.

##### Quadriceps Strength

During hospitalization, quadriceps strength was assessed every day by asking the patients to flex their hip and knee. Scoring was done as follows: 0 point, no muscle contraction; 1 point, muscle contraction but no joint movement; 2 points: joint movement but no gravity resistance; 3 points, gravity resistance; 4 points: gravity resistance and partial counterforce resistance; and 5 points: normal joint function. Quadriceps strength was also assessed at 3 months after surgery.

##### Time to First Straight Leg Rise

After surgery, the time to first straight leg rise was recorded.

##### Daily Ambulation Distance

During hospitalization, the patient was asked to walk the longest distance possible in one attempt every day. The distance was measured and recorded.

##### The Duration of Hospitalization

The duration of hospitalization was recorded. Discharge criteria included adequate pain control using oral medication, independent transfer, ambulation of at least 200 feet, and the ability to climb stairs.

##### Oxford Hip Score

The Oxford hip score was used to evaluate postoperative recovery of hip function. The Oxford hip score system mainly includes postoperative pain and function recovery of the hip. The score ranges from 0 to 48, with a higher score suggesting better outcome.

##### UCLA Activity Level Rating

The UCLA activity level rating was used to assess the limb function and the activity level of patients, with a scale of 1–10, where 1 indicates that the individual is wholly inactive (dependent on others and cannot leave residence) and 10 indicates regular participation in impact sports, such as jogging, tennis, skiing, acrobatics, ballet, heavy labor, or backpacking.

All the above outcomes were evaluated by the same investigator and this outcomes assessor was unaware of the treatment group.

##### Postoperative Complications

The occurrence of postoperative complications was recorded by another investigator, including nausea, vomiting, postoperative chronic pain (VAS score ≥4 during daily activities at 3 months after surgery), LFCN dysesthesia (numbness or bothersome numbness in the region innervated by LFCN), additional nerve damage, VTE, postoperative infection, and falls after surgery.

### Statistical Analysis

All data are presented as mean and standard deviation, unless indicated otherwise. The normality of data was analyzed using histograms and Q‐Q plots. Intergroup differences in continuous, normally distributed data were assessed using Student's *t*‐test. Differences in categorical data were assessed using Pearson's *c* ^2^‐test or Fisher's exact probabilities test. Differences in ordinal, skewed data were assessed using the Mann–Whitney *U*‐test. All statistical analyses were performed using SPSS 25.0 (IBM, Chicago, IL, USA). Differences were considered statistically significant when *P* < 0.05.

## Results

### Patient Characteristics

Based on the date of admission, 83 patients were included in the control group and 72 in the nerve block group. Follow‐up data (up to 3 months after surgery) was not available for eight patients in the control group and five patients in the nerve block group. After excluding these patients, we analyzed data from 75 patients in the control group and 67 in the nerve block group. In the control group, 43 patients received the longitudinal incision and 32 received the “bikini” incision; the corresponding numbers in the nerve block group were 36 and 31.

The two surgery groups did not differ significantly in age, gender, BMI, surgery side, incision type, or ASA status (Table 1). Simultaneously, there were no significant differences in preoperative VAS pain scores, quadriceps strength, Oxford hip score, or UCLA activity level rating between the two groups. The same results were observed after stratifying patients by incision type (Tables 2 and 3). In the two subgroups, patients who did and did not receive LFCNB + IHINB did not differ significantly in age, gender, BMI, surgery side, incision type, ASA status, preoperative VAS pain score, quadriceps strength, Oxford hip score, or UCLA activity level rating.

**TABLE 1:** Clinical characteristics of total hip arthroplasty patients

Outcome	Control group (*n* = 75)	Nerve block group (*n* = 67)	*P‐*value
Age (years)	54.7 (10.9)	55.9 (12.6)	0.540*
Gender (M/F)	41/34	36/31	0.911†
BMI (kg/m^2^)	24.3 (2.6)	24.2 (2.3)	0.817*
Surgery side (right/left)	43/32	35/32	0.542†
Incision type (longitudinal/ bikini)	43/32	36/31	0.666†
ASA status (I/II/III)	13/45/17	9/44/14	0.853‡
Preoperative measures			
VAS pain scores	4.6 (0.9)	4.7 (1.0)	0.815‡
Quadriceps strength	4.9 (0.3)	4.9 (0.3)	0.991‡
Oxford hip score	26.3 (7.4)	26.6 (7.1)	0.795‡
UCLA activity level rating	4.0 (0.9)	3.9 (0.9)	0.556‡
Morphine consumption (mg)			
Postoperative day 1	14.1 (7.0)	9.9 (4.8)	<0.001‡
Postoperative day 2	3.5 (4.8)	3.3 (5.0)	0.724‡
Total	17.6 (7.3)	13.1 (7.6)	<0.001‡
Quadriceps strength			
Postoperative day 1	3.6 (0.7)	3.6 (0.7)	0.760‡
Postoperative day 2	4.2 (0.5)	4.3 (0.)	0.220‡
3 months	4.9 (0.3)	5.0 (0.2)	0.391‡
Daily mobilization (m)			
Postoperative day 1	16.9 (8.1)	21.7 (8.1)	<0.001‡
Postoperative day 2	32.7 (13.0)	39.3 (10.5)	0.001‡
QoR‐15 score			
Postoperative day 1	94.6 (6.6)	100.9 (7.3)	<0001‡
Postoperative day 2	107.4 (7.2)	108.1 (6.4)	0.516‡
Time to first straight leg raise (h)	9.9 (3.9)	7.7 (3.2)	<0.001‡
Oxford hip score			
1 month	32.3 (4.2)	32.2 (4.6)	0.928‡
3 months	42.3 (3.9)	42.1 (4.2)	0.928‡
UCLA activity level rating			
1 month	4.7 (0.8)	4.6 (0.7)	0.419‡
3 months	6.2 (0.6)	6.2 (0.5)	0.864‡
Postoperative hospitalization (h)	57.3 (15.4)	50.9 (10.1)	0.030‡
Postoperative complications (n, %)			
Nausea	24 (32.0%)	16 (23.9%)	0.283†
Vomiting	14 (18.7%)	9 (13.4%)	0.398†
Chronic pain	9 (12.0%)	4 (6.0%)	0.214†
LFCN dysesthesia	11 (14.7%)	10 (14.9%)	0.965†
Venous thrombotic events	2 (2.7%)	2 (3.0%)	1.000†
Additional nerve damage	0 (0%)	0 (0%)	
Infection	0 (0%)	0 (0%)	
Falls after surgery	0 (0%)	0 (0%)	

Values are mean (SD), number of cases or number of cases (percentage).

ASA, American Society of Anesthesiologists; BMI, body mass index; F, female; LFCN lateral femoral cutaneous nerve; M, male; QoR‐15 score, quality of recovery score; VAS, visual analogue scale.

* Student's *t*‐test.

† Pearson's c^2^‐test.

‡ Mann–Whitney *U*‐test.

**TABLE 2:** Clinical characteristics of total hip arthroplasty patients who underwent longitudinal incision

Outcome	Control group (*n* = 43)	Nerve block group (*n* = 36)	*P*‐value	
Age (years)	55.0 (12.5)	56.3 (13.7)	0.656*
Gender (M/F)	25/18	19/17	0.633†	
BMI (kg/m^2^)	23.8 (2.5)	24.0 (2.1)	0.682*	
Surgery side (right/left)	25/18	19/17	0.633†	
ASA status (I/II/III)	6/28/9	5/24/7	0.911‡	
Preoperative measures				
VAS pain scores	4.8 (0.8)	4.9 (1.0)	0.641‡	
Quadriceps strength	4.9 (0.3)	4.9 (0.4)	0.765‡	
Oxford hip score	25.8 (7.0)	25.4 (7.1)	0.840‡	
UCLA activity level rating	3.8 (0.8)	3.8 (0.8)	0.721‡	
Morphine consumption (mg)				
Postoperative day 1	13.5 (6.9)	8.9 (4.0)	0.001‡	
Postoperative day 2	3.7 (4.9)	3.3 (5.3)	0.603‡	
Total	17.2 (7.7)	12.2 (7.6)	0.002‡	
Quadriceps strength				
Postoperative day 1	3.6 (0.7)	3.7 (0.6)	0.416‡	
Postoperative day 2	4.2 (0.5)	4.3 (0.6)	0.557‡	
3 months	5.0 (0.2)	4.9 (0.2)	0.856‡	
Daily mobilization (m)				
Postoperative day 1	16.3 (8.1)	19.0 (6.4)	0.008‡	
Postoperative day 2	31.9 (13.8)	38.3 (11.1)	0.018‡	
QoR‐15 score				
Postoperative day 1	95.1 (6.7)	103.1 (7.2)	<0.001‡	
Postoperative day 2	105.5 (6.6)	108.2 (6.7)	0.064‡	
Time to first straight leg raise (h)	9.9 (4.2)	7.7 (3.7)	0.004‡	
Oxford hip score				
1 month	32.8 (4.1)	31.8 (4.8)	0.321‡	
3 months	43.1 (3.4)	41.4 (4.4)	0.085‡	
UCLA activity level rating				
1 month	4.6 (0.8)	4.7 (0.6)	0.537‡	
3 months	6.2 (0.7)	6.2 (0.5)	0.831‡	
Postoperative hospitalization (h)	59.2 (16.6)	52.0 (11.7)	0.173‡	
Postoperative complications (n, %)				
Nausea	14 (32.6%)	8 (22.2%)	0.307†	
Vomiting	7 (16.3%)	5 (13.9%)	0.768†	
Chronic pain	4 (9.3%)	2 (5.6%)	0.842†	
LFCN dysesthesia	6 (14.0%)	8 (22.2%)	0.338†	
Venous thrombotic events	1 (2.3%)	0 (0.0%)	1.000§	

Values are mean (SD), number of cases or number of cases (percentage).

ASA, American Society of Anesthesiologists; BMI, body mass index; F, female; LFCN, lateral femoral cutaneous nerve; M, male; QoR‐15 score, quality of recovery score; VAS, visual analogue scale.

* Student's *t*‐test.

† Pearson's c^2^‐test.

‡ Mann–Whitney *U*‐test.

§ Fisher's exact probabilities test.

**TABLE 3:** Clinical characteristics of total hip arthroplasty patients who underwent “bikini” incision

Outcome	Control group (*n* =32)	Nerve block group (*n* =31)	*P*‐value
Age (years)	54.3 (8.4)	55.4 (11.4)	0.654*
Gender (M/F)	16/16	17/14	0.701†
BMI (kg/m^2^)	25.0 (2.5)	24.5 (2.5)	0.391*
Surgery side (right/left)	18/14	16/15	0.712†
ASA status (I/II/III)	7/17/8	4/20/7	0.708‡
Preoperative measures			
VAS pain scores	4.3 (0.9)	4.4 (0.9)	0.954‡
Quadriceps strength	4.9 (0.3)	4.9 (0.3)	0.724‡
Oxford hip score	27.0 (7.8)	27.9 (6.9)	0.762‡
UCLA activity level rating	4.2 (0.8)	3.9 (0.9)	0.208‡
Morphine consumption (mg)			
Postoperative day 1	15.0 (7.2)	11.0 (5.4)	0.006‡
Postoperative day 2	3.1 (4.7)	3.2 (4.8)	0.932‡
Total	18.1 (6.9)	14.2 (7.6)	0.011‡
Quadriceps strength			
Postoperative day 1	3.8 (0.7)	3.6 (0.7)	0.184‡
Postoperative day 2	4.1 (0.6)	4.3 (0.5)	0.241‡
3 months	4.9 (0.3)	5.0 (0.2)	0.177‡
Daily mobilization (m)			
Postoperative day 1	17.7 (8.2)	24.8 (8.8)	<0.001‡
Postoperative day 2	33.8 (11.8)	40.3 (9.7)	0.023‡
QoR‐15 score			
Postoperative day 1	93.9 (6.6)	98.4 (6.6)	0.009‡
Postoperative day 2	109.9 (7.2)	108.1 (6.3)	0.175‡
Time to first straight leg raise (h)	9.8 (3.5)	7.6 (2.5)	0.005‡
Oxford hip score			
1 month	31.7 (4.4)	32.6 (4.4)	0.189‡
3 months	41.3 (4.3)	42.9 (3.9)	0.094‡
UCLA activity level rating			
1 month	4.8 (0.9)	4.5 (0.7)	0.078‡
3 months	6.2 (0.5)	6.1 (0.5)	0.619‡
Postoperative hospitalization (h)	54.6 (13.6)	49.6 (7.8)	0.107‡
Postoperative complications (n, %)			
Nausea	10 (31.3%)	8 (25.8%)	0.633†
Vomiting	7 (21.9%)	4 (12.9%)	0.348†
Chronic pain	5 (15.6%)	2 (6.5%)	0.449†
LFCN dysesthesia	5 (15.6%)	2 (6.5%)	0.449†
Venous thrombotic events	1 (3.1%)	2 (6.5%)	0.978†

Values are mean (SD), number of cases or number of cases (percentage).

ASA, American Society of Anesthesiologists; BMI, body mass index; F, female; LFCN, lateral femoral cutaneous nerve; M, male; QoR‐15 score, quality of recovery score; VAS, visual analogue scale.

* Student's *t*‐test.

† Pearson's c^2^‐test.

‡ Mann–Whitney *U*‐test.

### Postoperative Outcomes

#### Postoperative Morphine Consumption

Regardless of incision type, patients who received LFCNB + IHINB showed significantly lower postoperative morphine consumption within the first 24 h and lower total morphine consumption than the control group (Table 1). Similar results were observed when patients were stratified by incision type (Tables 2 and 3). In the two subgroups, patients who received LFCNB + IHINB had significantly lower postoperative morphine consumption within the first 24 h and lower total morphine consumption.

#### Postoperative Pain

**Fig. 5:**
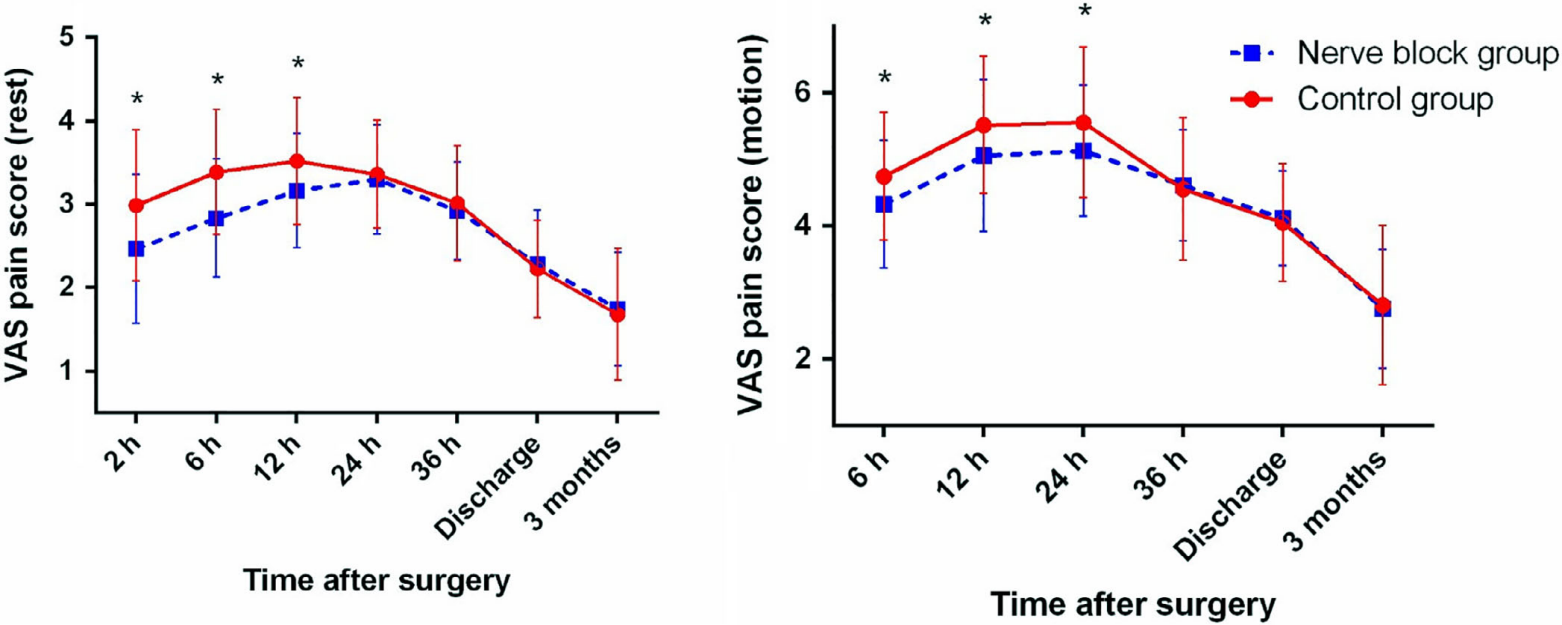
Average postoperative visual analogue scale (VAS) pain scores for all patients. Error bars indicate the standard deviation of the mean. *P < 0.05.

**Fig. 6:**
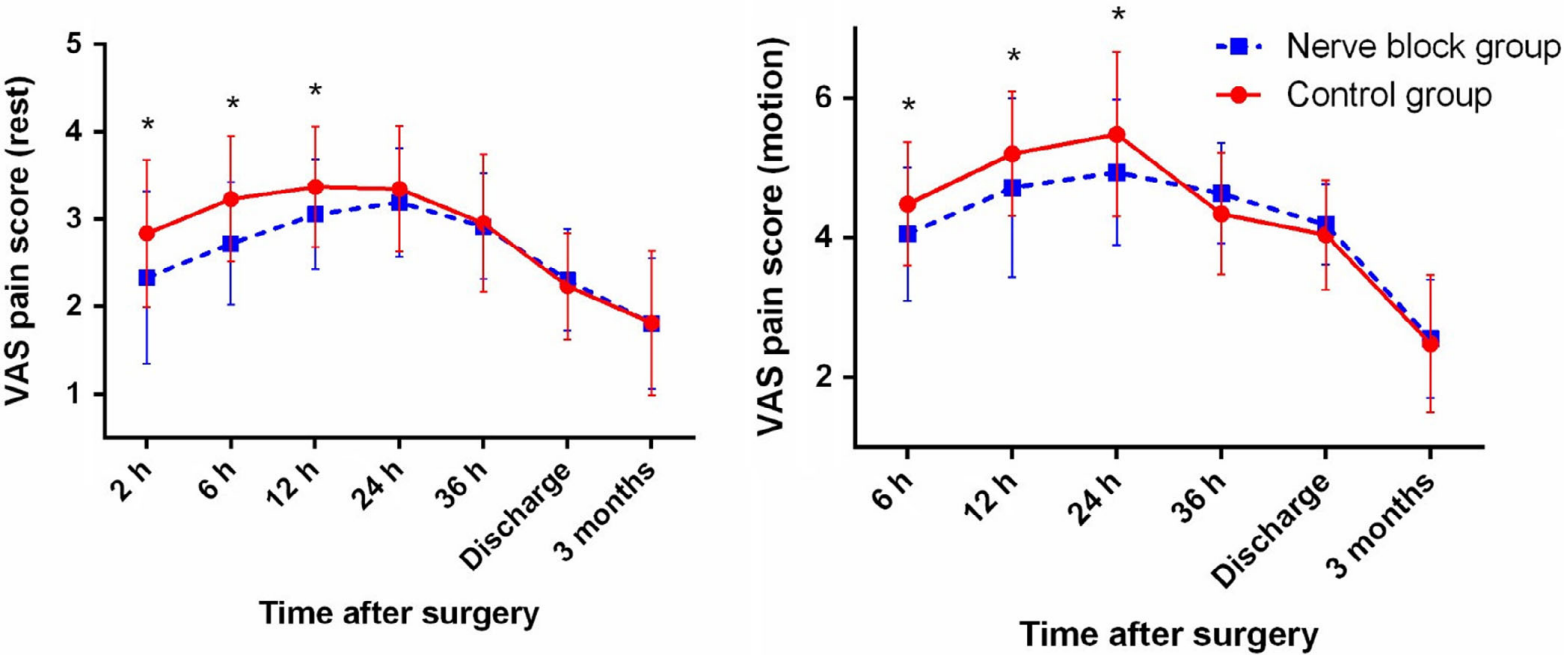
Average postoperative visual analogue scale (VAS) pain scores of patients who received traditional longitudinal incisions. Error bars indicate the standard deviation of the mean. *P < 0.05.

**Fig. 7:**
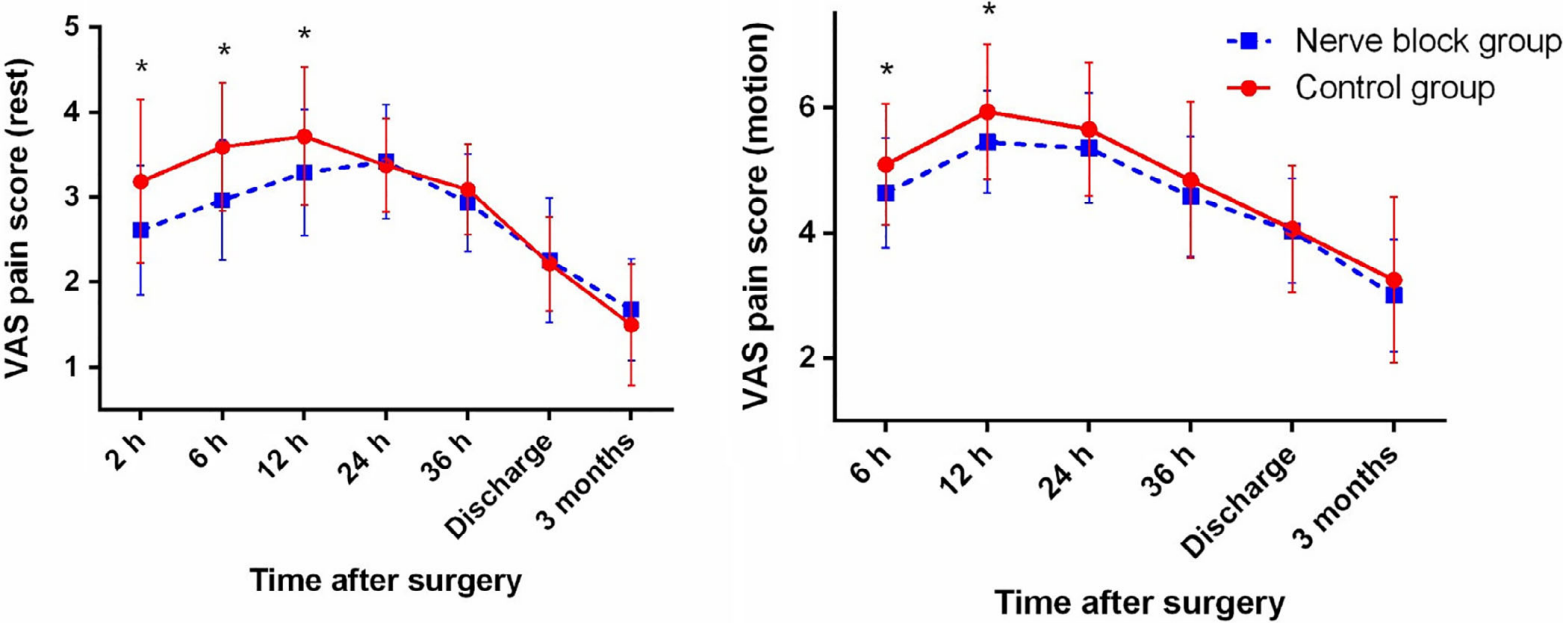
Average postoperative visual analogue scale (VAS) pain scores of patients who received bikini incisions. Error bars indicate the standard deviation of the mean. *P < 0.05.

Regardless of incision type, patients who received LFCNB + IHINB had significantly lower resting VAS scores within 12 h after surgery and significantly lower VAS scores during motion within 24 h after surgery than the control group (Fig. 5). When patients were stratified by incision type, similar results were observed in the longitudinal incision subgroup (Fig. 6). In the longitudinal incision subgroup, patients who received LFCNB + IHINB had significantly lower resting VAS scores within 12 h after surgery and significantly lower VAS scores during motion within 24 h after surgery. Difference was observed in the “bikini” incision subgroup, where patients who received nerve blocks had significantly lower resting VAS scores within 12 h after surgery and had significantly lower VAS scores during motion within 12 h after surgery but not at 24 h after surgery (Fig. 7).

#### Postoperative Recovery Quality

Regardless of incision type, patients who received LFCNB + IHINB had significantly better QoR‐15 scores on postoperative day 1 (Table 1). Similar results were observed when patients were stratified by incision type (Tables 2 and 3). In the two subgroups, patients who received LFCNB + IHINB had significantly better QoR‐15 scores on postoperative day 1.

### Postoperative Functional Recovery

#### Quadriceps Strength

Regardless of incision type, patients in the two surgery groups showed no significant differences in quadriceps strength on postoperative days 1 and 2, and at 3‐month outpatient follow‐up (Table 1). Similar results were observed when patients were stratified by incision type (Tables 2 and 3). In the two subgroups, patients who did and did not received LFCNB + IHINB did not differ significantly in quadriceps strength on postoperative days 1 and 2, and at 3‐month outpatient follow‐up.

#### Time to First Straight Leg Rise

Regardless of incision type, patients in the nerve block group completed their first straight leg rise significantly earlier than the control group (Table 1). Similar results were observed when patients were stratified by incision type (Tables 2 and 3). In the two subgroups, patients who received LFCNB + IHINB completed their first straight leg rise significantly earlier.

#### Daily Ambulation Distance

Regardless of incision type, patients in the nerve block group had significantly longer ambulation distances on postoperative days 1 and 2 than those in the control group (Table 1). Similar results were observed when patients were stratified by incision type (Tables 2 and 3). In the two subgroups, patients who received LFCNB + IHINB had significantly longer ambulation distances on postoperative days 1 and 2.

#### Duration of Hospitalization

Regardless of the incision type, patients in the nerve block group were hospitalized for significantly shorter time than those in the control group (Table 1). When patients were stratified by incision type, the duration of hospitalization was similar between the nerve block and control groups (Tables 2 and 3).

#### Oxford Hip Score

Regardless of incision type, the two groups showed no significant differences in Oxford hip score at 1‐month and 3‐month outpatient follow‐up (Table 1). Similar results were observed when patients were stratified by incision type (Tables 2 and 3). In the two subgroups, patients who did and did not receive LFCNB + IHINB did not differ significantly in Oxford hip score at 1‐month and 3‐month outpatient follow up.

#### UCLA Activity Level Rating

Regardless of incision type, the two groups showed no significant differences in UCLA activity level rating at 1‐month and 3‐month outpatient follow up (Table 1). Similar results were observed when patients were stratified by incision type (Tables 2 and 3). In the two subgroups, patients who did and did not receive LFCNB + IHINB did not differ significantly in UCLA activity level rating at 1‐month and 3‐month outpatient follow up.

#### Postoperative Complications

There was no significant difference between the two control and neural block groups in the incidence of postoperative complications, including nausea, vomiting, postoperative chronic pain, LFCN dysesthesia, or VTE (Table 1). None of the patients suffered from additional nerve damage, postoperative infection, or falls. Incision type also had no influence on postoperative complications (Tables 2 and 3). In the two subgroups, patients who did and did not receive LFCNB + IHINB did not differ significantly in the incidence of postoperative nausea, vomiting, chronic pain, LFCN dysesthesia, or VTE.

## Discussion

This study evaluated the efficacy and safety of LFCNB and IHINB for pain management after THA *via* the DAA. Our results suggest that, compared to the use of PIA alone, a combination of LFCNB and IHINB along with PIA can provide early‐stage postoperative pain relief, reduce postoperative morphine consumption, and accelerate functional recovery without increasing the risk of postoperative complications. However, these peripheral nerve blocks may not have a significant effect on long‐term outcomes.

With the enhanced recovery after surgery approach, THA patients should begin functional exercises as soon as possible^6^, ^29^. Therefore, reducing postoperative pain is important because it may seriously hinder and delay functional recovery and the ability of the patient to exercise^30^. PIA is a common part of the analgesic regimen for THA, but its efficacy in postoperative pain management is debated^4^, ^31^, ^32^. A sizeable proportion of patients who receive PIA at our institution report wound pain after THA *via* the DAA and studies have demonstrated that wound pain may play a role in pain after THA^14^, ^22^, ^23^. We hypothesized that a combination of peripheral nerve blocks (LFCNB and IHINB) along with PIA may provide a satisfactory analgesic effect for these patients because both the longitudinal and “bikini” incisions used during THA *via* the DAA are located in the area supplied by the iliohypogastric/ilioinguinal and the lateral femoral cutaneous nerve. Both nerve blocks are purely sensory blocks for the lower limbs.

An important goal of recovery after undergoing arthroplasty is excellent postoperative analgesia with minimal opioid consumption^33^, ^34^. In our study, we found that postoperative total morphine consumption was significantly lower in patients treated with LFCNB and IHINB. This should translate to a lower risk of opioid‐related adverse events, such as postoperative nausea and vomiting. Although the nerve block group experienced fewer incidents of nausea and vomiting, this was not significantly different from the control group. Based on the small sample in our study, we do not have enough evidence to fully understand the influence of these nerve blocks on opioid‐related adverse events. Our results also indicate that the positive analgesic effect provided by a combination of the nerve blocks and PIA disappeared over time: from postoperative day 2, there was no significant difference in VAS pain scores from the control group. This suggests that the local anesthetics used in this study do not provide a long‐lasting analgesic effect. Future studies should consider changing the composition of local anesthetics to prolong the effects of the nerve blocks and postoperative analgesia.

In addition to the VAS pain scores, patients treated with a combination of LFCNB and IHINB along with PIA had significantly better QoR‐15 scores on postoperative day 1 than patients treated with PIA alone. The QoR‐15 score provides a validated, comprehensive assessment of the quality of postoperative recovery^26^. Because recovery after surgery and anesthesia depend on patient, surgical, and anesthetic characteristics, as well as the incidence rate of complications, further research should be conducted on specific aspects of recovery that show improvement when a combination of nerve blocks (LFCNB and IHINB) is used with PIA.

Consistent with better VAS pain scores and QoR‐15 scores, we observed better early‐stage functional recovery during hospitalization in the neural block group and the patients had significantly shorter hospital stay. These results are consistent with the idea that better early pain relief can translate to better functional recovery. At the same time, the two groups showed no significant difference in quadriceps strength, suggesting that these two nerve blocks do not reduce muscle strength in the lower limbs. However, pain and function assessed using the Oxford hip score and the UCLA activity level rating at 1‐month and 3‐month outpatient follow up were similar for both groups. Further studies with large sample sizes are required to validate and extend our results, as well as explore how to improve the long‐term outcomes of using LFCNB and IHINB along with PIA.

As for the safety of these two nerve blocks, we found that adding LFCNB and IHINB to PIA did not increase the incidence of postoperative complications in our small sample study. Large sample sizes are still required to validate our results.

We observed advantages of LFCNB and IHINB regardless of whether the patient underwent a longitudinal or “bikini” incision, except that hospitalization duration was similar for the nerve block and control groups. This should be verified in larger studies and the reasons should be explored.

This is the first study to propose the idea of using LFCNB and IHINB for postoperative analgesia in THA *via* the DAA. This study provides preliminary support for the use of these nerve blocks for postoperative pain management. However, our results should be interpreted with caution in light of several limitations. First, the sample size of our study is small, and studies with larger sample sizes are still required to validate our results. Second, lack of randomization in patient assignment to the nerve block or control group is another limitation of this study. Nevertheless, the two groups did not differ in clinicodemographic characteristics. The retrospective design is open to biases that would be reduced with a prospective randomized controlled design. Third, this study did not analyze long‐term outcomes and complications beyond 3 months after surgery. In future studies, long‐term outcomes and complications must be studied in detail with extensive follow up.

### Conclusion

Compared to PIA alone, ultrasound‐guided LFCNB and IHINB along with PIA is an effective strategy to improve short‐term pain relief, reduce morphine consumption, and accelerate early‐stage functional recovery after THA *via* the DAA, using both longitudinal and “bikini” incisions, without increasing the incidence of complications. Prospective studies with fewer confounding factors are needed to further explore the clinical benefits of this method.

## References

[1] Skinner HB , Shintani EY . Results of a multimodal analgesic trial involving patients with total hip or total knee arthroplasty. Am J Orthop (Belle Mead NJ), 2004, 33: 85–92. 15005598

[2] Wylde V , Rooker J , Halliday L , Blom A . Acute postoperative pain at rest after hip and knee arthroplasty: severity, sensory qualities and impact on sleep. Orthop Traumatol Surg Res, 2011, 97: 139–144. 21388906 10.1016/j.otsr.2010.12.003

[3] Burns LC , Ritvo SE , Ferguson MK , Clarke H , Seltzer Z , Katz J . Pain catastrophizing as a risk factor for chronic pain after total knee arthroplasty: a systematic review. J Pain Res, 2015, 8: 21–32. 25609995 10.2147/JPR.S64730 PMC4294690

[4] Andersen L , Kehlet H . Analgesic efficacy of local infiltration analgesia in hip and knee arthroplasty: a systematic review. Br J Anaesth, 2014, 113: 360–374. 24939863 10.1093/bja/aeu155

[5] Højer Karlsen AP , Geisler A , Petersen PL , Mathiesen O , Dahl JB . Postoperative pain treatment after total hip arthroplasty: a systematic review. Pain, 2015, 156: 8–30. 25599296 10.1016/j.pain.0000000000000003

[6] Soffin EM , YaDeau JT . Enhanced recovery after surgery for primary hip and knee arthroplasty: a review of the evidence. Br J Anaesth, 2016, 117: iii62–iii72. 27940457 10.1093/bja/aew362

[7] Pepper AM , Mercuri JJ , Behery OA , Vigdorchik JM . Total hip and knee arthroplasty perioperative pain management: what should be in the cocktail. JBJS Rev, 2018, 6: e5. 10.2106/JBJS.RVW.18.00023 30562208

[8] Johnson RL , Kopp SL , Hebl JR , Erwin PJ , Mantilla CB . Falls and major orthopaedic surgery with peripheral nerve blockade: a systematic review and meta‐analysis. Br J Anaesth, 2013, 110: 518–528. 23440367 10.1093/bja/aet013 PMC3600943

[9] Sürücü HS , Tanyeli E , Sargon MF , Karahan ST . An anatomic study of the lateral femoral cutaneous nerve. Surg Radiol Anat, 1997, 19: 307–310. 9413078 10.1007/BF01637599

[10] Grothaus MC , Holt M , Mekhail AO , Ebraheim NA , Yeasting RA . Lateral femoral cutaneous nerve: an anatomic study. Clin Orthop Relat Res, 2005, (437): 164–168. 10.1097/01.blo.0000164526.08610.97 16056045

[11] Doklamyai P , Agthong S , Chentanez V , *et al*. Anatomy of the lateral femoral cutaneous nerve related to inguinal ligament, adjacent bony landmarks, and femoral artery. Clin Anat, 2008, 21: 769–774. 18942079 10.1002/ca.20716

[12] Dimitropoulos G , Schaepkens van Riempst J , Schertenleib P . Anatomical variation of the lateral femoral cutaneous nerve: a case report and review of the literature. J Plast Reconstr Aesthet Surg, 2011, 64: 961–962. 21208836 10.1016/j.bjps.2010.11.020

[13] Thybo KH , Mathiesen O , Dahl JB , Schmidt H , Hägi‐Pedersen D . Lateral femoral cutaneous nerve block after total hip arthroplasty: a randomised trial. Acta Anaesthesiol Scand, 2016, 60: 1297–1305. 27426231 10.1111/aas.12764

[14] Thybo KH , Schmidt H , Hägi‐Pedersen D . Effect of lateral femoral cutaneous nerve‐block on pain after total hip arthroplasty: a randomised, blinded, placebo‐controlled trial. BMC Anesthesiol, 2016, 16: 21. 27006014 10.1186/s12871-016-0183-4 PMC4804512

[15] Okur O , Tekgul ZT , Erkan N . Comparison of efficacy of transversus abdominis plane block and iliohypogastric/ilioinguinal nerve block for postoperative pain management in patients undergoing inguinal herniorrhaphy with spinal anesthesia: a prospective randomized controlled open‐label study. J Anesth, 2017, 31: 678–685. 28616651 10.1007/s00540-017-2378-3

[16] Lin LC , Sun YC , Tseng KF , Chang RY , Leung HK . Comparison of preoperative and postoperative iliohypogastric ilioinguinal nerve block for pediatric herniorrhaphy patients. Ma Zui Xue Za Zhi, 1993, 31: 91–96. 7934692

[17] Wehbe SA , Ghulmiyyah LM , Dominique el KH , *et al*. Prospective randomized trial of iliohypogastric‐ilioinguinal nerve block on post‐operative morphine use after inpatient surgery of the female reproductive tract. J Negat Results Biomed, 2008, 7: 11. 19040739 10.1186/1477-5751-7-11 PMC2621114

[18] Hosalli V , Ayyanagouda B , Hiremath P , Ambi U , Hulkund SY . Comparative efficacy of postoperative analgesia between ultrasound‐guided dual transversus abdominis plane and Ilioinguinal/Iliohypogastric nerve blocks for open inguinal hernia repair: an open label prospective randomised comparative clinical trial. Indian J Anaesth, 2019, 63: 450–455. 31263296 10.4103/ija.IJA_153_19 PMC6573039

[19] Leunig M , Faas M , von Knoch F , Naal FD . Skin crease ‘bikini’ incision for anterior approach total hip arthroplasty: surgical technique and preliminary results. Clin Orthop Relat Res, 2013, 471: 2245–2252. 23412730 10.1007/s11999-013-2806-0 PMC3676627

[20] Leunig M , Hutmacher JE , Ricciardi BF , Impellizzeri FM , Rüdiger HA , Naal FD . Skin crease ‘bikini’ incision for the direct anterior approach in total hip arthroplasty: a two‐ to four‐year comparative study in 964 patients. Bone Joint J, 2018, 100‐B: 853–861. 10.1302/0301-620X.100B7.BJJ-2017-1200.R2 29954218

[21] Manrique J , Paskey T , Tarabichi M , Restrepo C , Foltz C , Hozack WJ . Total hip arthroplasty through the direct anterior approach using a bikini incision can be safely performed in obese patients. J Arthroplasty, 2019, 34: 1723–1730. 31003782 10.1016/j.arth.2019.03.060

[22] Ilchmann T , Gersbach S , Zwicky L , Clauss M . Standard transgluteal versus minimal invasive anterior approach in hip arthroplasty: a prospective, consecutive cohort study. Orthop Rev (Pavia), 2013, 5: e31. 24416475 10.4081/or.2013.e31 PMC3883072

[23] Repantis T , Bouras T , Korovessis P . Comparison of minimally invasive approach versus conventional anterolateral approach for total hip arthroplasty: a randomized controlled trial. Eur J Orthop Surg Traumatol, 2015, 25: 111–116. 24557411 10.1007/s00590-014-1428-x

[24] de Steiger RN , Lorimer M , Solomon M . What is the learning curve for the anterior approach for total hip arthroplasty? Clin Orthop Relat Res, 2015, 473: 3860–3866. 26394641 10.1007/s11999-015-4565-6 PMC4626490

[25] Hawker GA , Mian S , Kendzerska T , French M . Measures of adult pain: visual analog scale for pain (VAS pain), numeric rating scale for pain (NRS pain), McGill pain questionnaire (MPQ), short‐form McGill pain questionnaire (SF‐MPQ), chronic pain grade scale (CPGS), short Form‐36 bodily pain scale (SF‐36 BPS), and measure of intermittent and constant osteoarthritis pain (ICOAP). Arthritis Care Res (Hoboken), 2011, 63: S240–S252. 22588748 10.1002/acr.20543

[26] Stark PA , Myles PS , Burke JA . Development and psychometric evaluation of a postoperative quality of recovery score: the QoR‐15. Anesthesiology, 2013, 118: 1332–1340. 23411725 10.1097/ALN.0b013e318289b84b

[27] Dawson J , Fitzpatrick R , Carr A , Murray D . Questionnaire on the perceptions of patients about total hip replacement. J Bone Joint Surg Br, 1996, 78: 185–190. 8666621

[28] Zahiri CA , Schmalzried TP , Szuszczewicz ES , Amstutz HC . Assessing activity in joint replacement patients. J Arthroplasty, 1998, 13: 890–895. 9880181 10.1016/s0883-5403(98)90195-4

[29] Jakobsen TL , Kehlet H , Husted H , Petersen J , Bandholm T . Early progressive strength training to enhance recovery after fast‐track total knee arthroplasty: a randomized controlled trial. Arthritis Care Res (Hoboken), 2014, 66: 1856–1866. 25074397 10.1002/acr.22405

[30] Zhang Z , Yang Q , Xin W , Zhang Y . Comparison of local infiltration analgesia and sciatic nerve block as an adjunct to femoral nerve block for pain control after total knee arthroplasty: a systematic review and meta‐analysis. Medicine (Baltimore), 2017, 96: e6829. 28489762 10.1097/MD.0000000000006829 PMC5428596

[31] McCarthy D , Iohom G . Local infiltration analgesia for postoperative pain control following total hip arthroplasty: a systematic review. Anesthesiol Res Pract, 2012, 2012: 709531. 22829813 10.1155/2012/709531 PMC3398576

[32] Yin JB , Cui GB , Mi MS , *et al*. Local infiltration analgesia for postoperative pain after hip arthroplasty: a systematic review and meta‐analysis. J Pain, 2014, 15: 781–799. 24709160 10.1016/j.jpain.2014.03.002

[33] Baratta JL , Gandhi K , Viscusi ER . Perioperative pain management for total knee arthroplasty. J Surg Orthop Adv, 2014, 23: 22–36. 24641894 10.3113/jsoa.2014.0022

[34] Kampitak W , Tanavalee A , Ngarmukos S , Amarase C . Opioid‐sparing analgesia and enhanced recovery after Total knee arthroplasty using combined triple nerve blocks with local infiltration analgesia. J Arthroplasty, 2019, 34: 295–302. 30401559 10.1016/j.arth.2018.10.009

